# Selecting instruments for assessing psychological wellbeing in Afghan and Kurdish refugee groups

**DOI:** 10.1186/1756-0500-3-237

**Published:** 2010-09-08

**Authors:** Cheryl MR Sulaiman-Hill, Sandra C Thompson

**Affiliations:** 1 Centre for International Health, Curtin University, GPO Box U1987, Perth WA, Australia; 2 Winthrop Professor, Chair of Rural Health (University of Western Australia), Director, Combined Universities Centre for Rural Health, P.O. Box 109, Geraldton WA6531, Australia

## Abstract

**Background:**

Afghan and Iraqi refugees comprise nearly half of all those currently under United Nations protection. As many of them will eventually be resettled in countries outside the region of origin, their long term health and settlement concerns are of relevance to host societies, and will be a likely focus for future research. Since Australia and New Zealand have both accepted refugees for many years and have dedicated, but different settlement and immigration policies, a study comparing the resettlement of two different refugee groups in these countries was undertaken. The purpose of this article is to describe the instrument selection for this study assessing mental health and psychological well being with Afghan and Kurdish former refugees, in particular to address linguistic considerations and translated instrument availability. A summary of instruments previously used with refugee and migrant groups from the Middle East region is presented to assist other researchers, before describing the three instruments ultimately selected for the quantitative component of our study.

**Findings:**

The Kessler-10 Psychological Distress Scale (K10), General Perceived Self-Efficacy Scale (GPSE), and Personal Well-Being Index (PWI) all showed good reliability (Cronbach's alphas of 0.86, 0.89 and 0.83 respectively for combined language versions) and ease of use even for pre-literate participants, with the sample of 193 refugees, although some concepts in the GPSE proved problematic for a small number of respondents. Farsi was the language of choice for the majority of Afghan participants, while most of the Kurds chose to complete English versions in addition to Farsi. No one used Arabic or Turkish translations. Participants settled less than ten years were more likely to complete questionnaires in Farsi. Descriptive summary statistics are presented for each instrument with results split by gender, refugee group and language version completed.

**Conclusion:**

This paper discusses instrument selection for Farsi and Arabic speaking refugee participants from the Middle East and Afghanistan, concluding that the Kessler-10, GPSE scale and PWI were suitable for use with these groups. Suitable language translations are freely available. Our experience with these instruments may help inform other studies with these vulnerable groups.

## Background

Worldwide, conflict situations and the resultant number of refugees continues to increase, with over 40 million forcibly displaced people recorded in 2008 [1], and nearly half of these originally coming from Iraq or Afghanistan. As many will eventually be resettled elsewhere, their long term health and settlement concerns are of continuing relevance, providing a likely focus for research due to a high prevalence of mental health problems among these groups[2]. Since Australia and New Zealand have both accepted refugees for many years and have dedicated but distinctly different settlement policies, a study was proposed to compare the resettlement of two discrete refugee groups, Afghans and Kurds resettled and living in Christchurch or Perth, by assessing their health and subjective well-being (SWB). The main findings of the study will be reported separately. However, as a major challenge involved the selection of standardised, validated instruments in appropriate languages to measure the outcomes of interest with these ethnic groups, the aim of this article is to describe the instrument selection criteria, taking into consideration language requirements and a review of previous instruments used with refugees and groups from Afghanistan and the Middle East region. The three instruments eventually used for the study will be briefly outlined, and participant language preferences, instrument reliability and baseline descriptive statistics for the 193 former refugees presented to assist other researchers planning studies or working in this area.

## Method

### Study design

A mixed methods approach was used, combining qualitative interview data on resettlement experiences with quantitative assessment of psychological distress, general perceived self efficacy and subjective well being in a sample of adult Kurdish and Afghan former refugees settled for up to twenty years. The study was approved by the Human Research Ethics Committee, Curtin University of Technology in Perth.

### Statistical analysis

Quantitative data was analysed using SPSS 12.0 (SPSS Inc.). Frequency distributions for each language version by demographic variables and baseline descriptive statistics were calculated for each instrument. Kruskal-Wallis and Mann-Whitney U tests were performed to assess differences between groups of variables. Significant results from the Kruskal-Wallis test were further analysed by pair wise comparison using the Mann-Whitney test and the Bonferroni correction to determine significance level. Cronbach's alpha was calculated to assess reliability of the instruments.

### Criteria for Instrument selection

Language considerations were a major concern, as although many former refugees, especially those who have been settled for several years have a good understanding of English, there is not only an ethical imperative to ensure participants fully understand the implications and reason for the research, but because study validity could be compromised if instrument concepts are poorly understood. For this reason, the availability of pre-translated instruments in appropriate languages for the selected target populations was a key criterion in their selection. Instruments needed to be available in Farsi (Persian) as it is a national language of Afghanistan (Dari) and is also understood by many Kurdish refugees, as well as Arabic and English. No instruments were identified in any of the Kurdish dialects, however, as most Kurds have been educated in relevant the state languages, Farsi and Arabic were considered a compromise choice. In addition to availability in appropriate languages, we also required questionnaires to report adequate validity and reliability with comparable populations, measure the constructs of interest, and ideally have comparative national or local population data sets available.

Because of our Afghan and Kurdish focus, articles describing research with refugee and migrant participants from the Middle East were reviewed to identify instruments that had been selected by other authors (summarised in Table 1). This revealed no clear consensus on which to base the choice of instruments, as many authors did not discuss language details.

**Table 1:** Published studies of health and wellbeing in Afghan and Middle Eastern refugees and migrants

Author	Instruments	Outcome variables	Study participants
Ahmad et al [22]	-PTSS-C -CPTSD-RI	PTSD stress symptoms in traumatized children	Kurdish children in Iraq and Sweden & Swedish children
Casimiro,Hancock & Northcote [23]	Qualitative	Exploring resettlement issues during first five years	80 Muslim women (35 Iraqi, 34 Sudanese, 11 Afghan) in Perth, WA
Gerritsen et al [24]	-MOS -SF36 -HTQ -HSCL25	General health, PTSD, depression & anxiety	178 refugees & 262 asylum seekers (Iranian, Afghan & Somali) in the Netherlands
Ghazinour, Richter & Eisemann [25]	-WHOQOL100 -SOC -CRI -ISSI -BDI -SCL90R	Sense of coherence, coping resources & social support	100 Iranian refugees settled in Sweden
Gilgen et al [26]	-EMIC	Health interview for common health problems	36 Bosnian, 62 Turkish/Kurdish & 48 Swiss internal migrants in Switzerland
Hafshejani [27]	-PDS -LRI	PTSD & meaning in life	59 Iranian & Afghan males who have experienced war, now in Sydney
Hosin et al [28]	-GHQ30 -CBCL	Psychological wellbeing & adjustment	61 Arab & Kurdish families (including 162 children) in London
Husni et al [29]	-CAS	Satisfaction ratings of personal safety, health, employment, food, financial security, social life & entertainment	54 Kurdish refugees, 29 living in the UK & 25 in Canada
Ichikawa, Nakahara & Wakai [30]	-HSCL25 -HTQ	Assessment of post-migration detention on mental health	55 Afghan asylum seekers in Japan
Koehn [31]	Qualitative	Transnational competence, asylum seeker & clinician perspectives	41 asylum seekers from former Soviet Union, former Yugoslavia, Kurdish areas of Middle East & Somalia in Finnish reception centres
Omeri, Lennnings & Raymond [32]	Qualitative	Access, use & appropriateness of mental & physical health services	25 general & 13 key informant Afghan immigrants & refugees in NSW, Australia
Ross-Sheriff [33]	Qualitative	Women's experiences before & during war & exile	60 repatriated Afghan refugee women in Kabul
Sondergaard, Ekblad & Theorell [34]	-LED	Life events, ongoing difficulties & self reported health	86 refugees from Iraq (Arabic & Sorani speakers) in Stockholm
Taloyan et al [35]	Swedish National Survey & Level of Living Survey data	Association between ethnicity, poor self reported health, psychological distress, sleeping difficulties & use of psychotropic drugs	Immigrant Kurdish men & native Swedish men living in Sweden

Abbreviations of Instruments: BDI = Beck Depression Inventory; CAS = Cernovsky's Assimilation Scale; CBCL = Child Behavioural Checklist (modified); CPTSD-RI = Child Posttraumatic Stress Disorder Reaction Index; CRI = Coping Resources Inventory; EMIC = Explanatory Model Interview Catalogue; GHQ12 = General Health Questionnaire 12; GHQ30 = General Health Questionnaire 30; HSCL25 = Hopkins Symptoms Checklist 25; HTQ = Harvard Trauma Questionnaire; ISSI = Interview Schedule of Social Interaction; LED = Life Events & Ongoing Difficulties; LRI= Life Regard Index; MOS = Medical Outcome Study; PDS = Posttraumatic Stress Diagnostic Scale; PTSS-C = Posttraumatic Stress Symptoms in Children; SCL90R = Symptom Checklist 90; SF36 = Short Form Health Survey 36; SOC = Sense of Coherence Scale; WHOQOL100 = WHO Quality of Life 100

Following extensive database and internet searching three instruments were selected for the study, with the format, scoring, website information and comparative data sources summarised in Table 2. All are freely available from the website links listed, in a selection of languages suitable for use with groups from the Middle East region.

**Table 2:** Summary of instrument characteristics

	KESSLER-10	GPSE SCALE	PERSONAL WELLBEING INDEX
**LANGUAGES** (suitable for groups from Middle East region)	-Arabic -Persian (Farsi) -Turkish	-Arabic -Persian (Farsi) -Turkish	-Arabic -Persian (Farsi)
**AVAILABILITY**	Website (free)	Website (free)	Website (free)
**FORMAT**	-5 point Likert scale -10 questions relating to psychological distress in previous four weeks -Optional 4 questions assessing degree of disability, e.g. number of days totally unable to cope, number of days activity is cut back, number of times health professional was consulted & how many times physical problems have been the cause of distress	-4 point Likert scale -10 questions rating how well each statement describes the participants response to a problem situation	-11 point Likert scale -8 questions relating to satisfaction with quality of life domains • Standard of living • Health • Life achievement • Personal relationships • Personal safety • Community belonging • Future security • Religion/spirituality -Satisfaction with life as a whole can be included as optional first question
**SCORING**	-Items scored between 1 (none of the time) & 5 (all of the time) -Missing values excluded -Sum of all items gives total score with range 10 (low risk) - 50 (severe risk of distress) -Optional questions are excluded from total score	-Items scored between 1 (not at all true) & 4 (exactly true) -Sum of all items gives total score between 10-40, or mean score can be used -Scores not calculated if more than 3 missing values	-Items scored between 0 (completely dissatisfied) & 10 (completely satisfied) -Screen to remove response sets -Convert to %scale maximum value -Analyse as separate variables or aggregate to give average score for subjective wellbeing
**COMPARATIVE DATA AVAILABLE**	Yes (NSW Population Health Survey 2007, Australian Bureau Statistics Health surveys, NZ Health survey 2006/07 - see websites http://www.health.nsw.gov.au, http://www.abs.gov.au, http://www.moh.govt.nz)	Yes (website below)	Yes (website below)

Instruments available for download from:

*Kessler-10*: http://www.healthtranslations.vic.gov.au/bhcv2/bhcht.nsf/PresentDetail?Open&s=Kessler_10_measure

*GPSE Scale*: http://userpage.fu-berlin.de/health/selfscal.htm

*Personal Wellbeing Index*: http://acqol.deakin.edu.au/instruments/index.htm

#### • Kessler-10 Psychological Distress Scale (K-10)

The Kessler-10 scale is a population screening tool for psychological distress and has been used in New Zealand and Australian National Health and state surveys[3,4]. The K-10 consists of ten questions designed to measure psychological distress over the previous four weeks, scored with five response categories on a Likert scale. The sum of all ten items gives a total score with a range from 10 to 50. Variations in cut off levels have been noted; however, the NZ and Australian health surveys use the following criteria: scores of 10-15.9 indicate a low risk of psychological distress; 16-21.9 indicates an individual may be experiencing moderate levels of distress consistent with a diagnosis of moderate depression and/or anxiety disorder; 22-29.9 suggest a high level of distress; and scores of 30 or more indicate the possibility of very high or severe levels of distress. An additional four questions, which do not contribute to the final score, are included to assess the impact or degree of disability associated with the identified level of distress. Only people scoring above the minimum are asked to complete these. The questionnaire has been translated into Farsi, Arabic, and Turkish and validated by the Transcultural Mental Health Centre in NSW, Australia.

One recent study assessed the psychometric properties of the instrument with Moroccan and Turkish respondents, concluding that it is a reliable and valid screening instrument for anxiety and depression among groups from the Middle East [5]. The K-10 compares favourably with diagnostic interviews (World Health Organization Composite International Diagnostic Interview (CIDI)) and also with the General Health Questionnaire-12 (GHQ-12) [6].

#### • General Perceived Self-Efficacy Scale (GPSE)

The GPSE aims to assess an individual's general sense of self-efficacy, reflecting their ability to cope with daily hassles and flexibility to adapt after experiencing stressful life events. It correlates positively with self-esteem and optimism and negatively with anxiety, depression and physical symptoms. Efficacy beliefs control levels of motivation and perseverance, resilience to adverse situations, and they impact on an individual's vulnerability to stress and depression, as well as influencing life choices [7]. Measurement of generalised self efficacy has been subject to debate, although recent studies have confirmed it as a global construct [8,9]. The scale consists of ten questions in which respondents rate how well each statement describes their approach to problem situations on a four point Likert scale. A sum score, with a range from 10 to 40 points, can be calculated by adding all responses, or alternatively a mean score may be used. Higher scores represent higher perceived self efficacy. If there are more than three missing values, scores are not calculated. The scale is available in 30 languages from the website listed in Table 2, which also provides links to comparative data sets.

#### • Personal Well-Being Index (PWI)

The Australian Unity Well-Being Index (Personal Well-Being Index) was selected to measure subjective wellbeing, through eight domains representing the first level deconstruction of the global question 'How satisfied are you with your life as a whole?'[10]. Domains comprise standard of living, health, life achievement, personal relationships, and personal safety, feeling part of the community, future security and spirituality/religion. The optional religion/spirituality domain was also included as this is an important component of subjective wellbeing for groups from the Middle East[11]. Questions are scored using an 11-point Likert scale with the anchors 0 'Completely dissatisfied' and 10 'Completely satisfied'. The domains can be analysed as separate variables, or aggregated to give an average percentage score representing subjective well being, with higher values representing greater satisfaction. The questionnaire has validated Farsi and Arabic versions showing acceptable sensitivity between different demographic groups, and normative datasets for Australian and international populations are also available for comparison from the developer's website (Table 2).

As all instruments were directly downloaded in suitable languages, further translation prior to use was not necessary. We offered participants pre-translated and validated Farsi and Arabic versions, in addition to English. Turkish versions of the K-10 and GPSE were also obtained (although not needed) due to anticipated variations in the demographic profile of Kurdish groups in Australia; however, the PWI was unavailable in this language.

We had participant information sheets outlining our study objectives and procedures, as well as consent forms professionally translated into Farsi and Sorani (Kurdish dialect) using a standard back-translation procedure for the benefit of participants and interpreters. During this process, the translators also checked the original translated instruments and prepared them so that they were well presented and their format was consistent.

Open-ended questions were included in the interview to provide qualitative feedback and personal perspectives on participants' resettlement experiences. These explored differences between home and host countries, resettlement difficulties and suggestions for improvement, assessment of support, and strategies for dealing with stress and ill health. Respondents were also given the opportunity to raise any other issues of concern or interest. Results for this will be reported separately.

### Participants

Participants were of Afghan or Kurdish ethnicity, 18 years or older at the time of the study, who had arrived in New Zealand or Australia as refugees or asylum seekers between 1988 and 2008 and were resident in either Perth or Christchurch at the time of the study. A link methodology sampling method was used to overcome some of the sampling challenges with socially invisible groups, including invisibility in national data sets (a particular issue for people of Kurdish ethnicity), difficulties with access and trust and concerns about research motives. Multiple access points into each of the four refugee groups helped reduce selection bias while improving representativeness of the sample [12,13]. At least six discrete snowball initiation points were used with each group, with a variety of people recruited from each entry point giving a good cross-section of each community.

## Results

The sample consisted of 193 former refugees living in Christchurch (n = 98) and Perth (n = 95), 47% were Afghan and 53% Kurdish; 48% of the sample was female. Participants' ages ranged from 18-70 years, with time since resettlement ranging from several months to 20 years. Although sixteen had been minors at the time of arrival, all except two were of school age, mostly teenagers and had clear recollections of the resettlement experience. Most (86%) of participants reported themselves as having functional English ability, with everyone settled over ten years being able to speak it. Despite this, many people still preferred to use Farsi versions of the questionnaires, as outlined in Table 3.

**Table 3:** Questionnaire language version selected by participants (n = 193)

Variable		English version		Farsi version		Test of significance		
		**n**	**%**	**n**	**%**	***U***	***Z***	***p***

Refugee group	Afghan Kurdish	32 81	36 79	58 22	64 21	2533.0	-6.046	0.000
Resettlement location	Christchurch Perth	45 68	46 72	53 27	54 28	3325.5	-3.608	0.000
Gender	Male Female	58 55	58 59	42 38	42 41	4467.0	-0.160	0.873
English ability	Speaks English No English	108 5	65 18.5	58 22	35 81.5	3477.0	-4.541	0.000
Time since resettlement	1-2 years 3-5 years 6-10 years 11-20 years	13 17 37 46	45 42 52 90	16 24 34 5	55 58 48 10	*Χ*^2^(1,192) = 23.10		0.000

There were significant differences in the language chosen between Afghans and Kurds (with Afghans more likely to choose Farsi versions), between those settled in Christchurch and Perth, and based on English language ability. Variations in language choice between locations were mainly due to differences in resettlement time; with participants in Perth settled longer overall. No gender differences were observed. The length of time settled influenced the language version completed. Using the Mann-Whitney test for each paired combination of categories and the Bonferroni correction (p = .008) significant results were observed between groups settled for between 1-2 years and 11-20 years (U = 404.0, Z = -4.406, p.000), 3-5 years and 11-20 years (U = 536.0, Z = -4.973, p.000), and 6-10 years and 11-20 years (U = 1121.0, Z = -4.431, p.000). This indicates that people settled 11 years or longer were more likely to complete English questionnaires.

Most participants' self-completed questionnaires in their chosen language, discussing responses to open-ended questions in English, with interpreter help as needed. No one requested Turkish copies and only a few people wanted Arabic copies as a cross reference for English. Likert formats proved easy to understand, even for pre-literate participants.

All instruments showed good reliability when tested with our data using separate English and Farsi versions, and also when combined with the entire sample of 193 participants, as shown in Table 4.

**Table 4:** Reliability testing of instruments - Cronbach's alpha

Instrument	English version (n = 113)	Farsi version (n = 80)	Total combined (n = 193)
	**α**	**α**	**α**
Kessler-10	0.86	0.86	0.86
GPSE	0.88	0.89	0.89
PWI	0.86	0.77	0.83

Descriptive findings from the study split by gender, refugee community, and the questionnaire language version completed, as well as the total score for the combined sample of 193 participants is presented in Table 5. A full analysis of the results will be reported separately (article in preparation). As shown, statistically significant differences in mean scores were noted by gender for each instrument, by refugee group for the PWI and between language versions for K-10 and GPSE.

**Table 5:** Participant descriptive statistics for each instrument

Variable		Kessler-10			GPSE			PWI (Subjective wellbeing)		
		
		Mean	SD	n	Mean	SD	n	Mean	SD	n
**Male**	(n = 100)	18.48	7.22	100	32.39	6.18	96	79.52	14.06	99
**Female**	(n = 93)	21.84	7.99	93	28.17	6.26	90	74.73	15.68	91
										
**Afghan**	(n = 90)	19.82	7.65	90	29.49	6.78	89	80.50	14.51	87
**Kurdish**	(n = 103)	20.34	7.90	103	31.12	6.27	97	74.45	14.93	103
										
**English version**	(n = 113)	18.75	6.66	113	32.00	5.46	110	75.56	15.55	112
**Farsi version**	(n = 80)	22.00	8.80	80	27.93	7.26	76	79.62	13.95	78
										
**Total score**	(n = 193)	20.10	7.77	193	30.34	6.55	186	77.22	15.01	190

		**TEST OF SIGNIFICANCE FOR EACH VARIABLE & INSTRUMENT**								

		***Kessler-10***				***GPSE***			***PWI***	
**Variable**		***U***	***Z***	***p***	***U***	***Z***	***p***	***U***	***Z***	***p***

**Gender**		3333.5	-3.401	0.001	2486.0	-5.007	0.000	3730.0	-2.046	0.041
**Refugee group**		4501.5	-0.345	0.730	3706.5	-1.666	0.096	3348.5	-2.999	0.003
**Language version**		3601.0	-2.408	0.016	2799.0	-3.833	0.000	3716.0	-1.749	0.080

Kessler-10 criteria: Low risk psychological distress 10-15.9; Moderate risk 16-21.9; High risk 22-29.9, Very high or severe risk 30-50.

GPSE: Aggregate scoring range 10-40 with higher scores suggesting higher levels of self efficacy.

PWI: Reporting subjective wellbeing as an aggregate percentage score, higher scores represent higher overall satisfaction.

## Discussion

Conflicts in the Middle East have led to large numbers of refugees from Afghanistan and Iraq who seek resettlement by the United Nations. Both conflict and globalisation have increased the movement of people between countries with very different cultural backgrounds, posing a number of methodological and ethical challenges for research with such groups. In particular, quantitative measures are needed, that allow comparison between groups and monitoring of trends related to resettlement.

The validity of a study using standardised instruments may be compromised if concepts are poorly understood by participants, so provision of validated instruments in suitable languages is necessary. Many instruments have been used in previous studies with refugee groups [14], and some such as the Harvard Trauma Questionnaire (HTQ) or Vietnamese Depression Scale were specifically developed for refugee research, however many of these instruments focus on pre-migration traumatic experiences or were developed only for use with specific groups. Hollifield and colleagues, [14] in a review of 183 articles describing trauma and health status in refugees, identified 12 specific refugee instruments but none met all their evaluation criteria for definition of purpose, construct definition, design, development and testing with refugee groups, nor were any of the instruments available in the published literature. Instruments such as the Hopkins Symptom Checklist (HSCL) and Beck Depression Inventory meet Hollifield's evaluation criteria, provide measures of general health status and have been adapted for use with forced migrants. These adapted instruments seemed a possibility for use, but many were not available in languages spoken by immigrants and refugees who come from the regions of our concern and have few traditional linkages to western academic or health care institutions. As the emphasis of our study was on a general overview of health and quality of life to reflect the daily realities associated with resettlement, specialised, diagnostic trauma instruments were not selected.

As described and summarised in Table 1, the next step in selecting suitable instruments was to identify instruments previously used with participants from the Middle East or Afghanistan. We included studies of refugees, asylum seekers or migrants living in resettlement countries. Of these, twenty instruments were used in ten quantitative studies, but no consensus on the suitability of different questionnaires emerged and it was unclear in many cases which language versions were used as this was rarely discussed, with the focus of most articles being on results and analysis. Of the well known instruments, the HSCL-25 and HTQ were used twice, and the General Health Questionnaire-30 (GHQ-30) was used once for assessment of mental health status. Although translations into Arabic and Farsi have been reported for some of these instruments, we could not locate them through searching published articles and the internet. In contrast, the instruments eventually chosen, although not specialised refugee tools, were freely available in translation, easy to find, did not require administration by specialist personnel and, with the exception of the GPSE, were commonly used or developed in Australasia so comparison with local national data sets was possible.

Ideally, translated instruments should have been validated with the community in question, or groups from similar cultural backgrounds, to reflect conceptual variations and different explanatory models [15,16]. If translations are not available, questionnaires need to undergo a standard translation/back translation process, taking care to ensure semantic and conceptual equivalence [17,18], avoidance of culturally sensitive material, and would need validation with each cultural group; a requirement beyond the scope of this and many other studies. The selection of previously translated versions of the instruments helped address these issues.

In practice, nearly 59 percent of study participants chose English language versions, with the remainder selecting Farsi questionnaires. We found significant differences between groups based on ethnic group, resettlement location, English language ability and resettlement time. People from Afghanistan were more likely to choose Farsi even many years after arrival, as it is their first language, while Kurdish respondents mainly chose English. No instruments were available in any of the Kurdish dialects; however most Kurds are educated in their state of origin languages, mainly Farsi, Arabic or Turkish and may not be literate in Kurdish, so this adds an extra layer of complexity and limitation for research with these groups. In a small number of cases, mainly for pre-literate participants, questionnaires were completed with interpreter assistance, so the language version used was dependent on them. Overall, participants in Perth had been settled longer, which accounted for some variation in English ability between the two locations and was also reflected in the language versions chosen.

Questionnaires showed good reliability (Table 4) when tested for each language version and with combined results. Amongst our participants, the PWI presented no problem for completion; however, a few people had trouble with some of the GPSE questions, with seven (three English, four Farsi) failing to complete the required number for inclusion. These asked participants to rate how well each statement described their approach to various situations, for example 'I am certain that I can accomplish my goals'. For those with strong religious beliefs (97 percent, mostly Muslim), the relevance of these concepts to their personal lives was not apparent. As one woman stated, "It doesn't matter what I think, God decides". Question 10 in the K-10 which asks if participants felt 'worthless' was culturally problematic for some Kurdish respondents as it challenged their ideal of human dignity, however they understood the reason for the question and responded accordingly. Despite these minor concerns, the instruments were easy to understand, with the format and Likert scales presenting no difficulties for participants, including those with limited literacy.

Researchers need to be cautious with interpretation of results, and aware that response biases have been reported in cross-cultural surveys with other instruments. In particular, acquiescent responses to personally relevant items have been more commonly observed in collectivist cultures [19,20]. Cut-off points for each instrument and population norms, preferably with existing result databases to allow meaningful comparisons and conclusions to be drawn, should also be available if possible. Determination of cut-off values normally involves comparison with other instruments or interviews as the 'gold standard' to assess the validity of the instrument and should ideally be determined for each cultural group surveyed. For example, high prevalence of anxiety and depression are commonly reported in Afghanistan, however, a comparison of standard mental health questionnaires with psychiatric interviews indicated differences in optimal cut-off points[21]. In particular, gender disparities have been noted, with recommended cut-off points lower than normal for men and higher for women, suggesting that some studies may have over or under-estimated prevalence rates respectively. Although it was beyond the scope of the present study to determine this, mean K-10 scores for females were 21.8 and for men 18.5, so even if these were adjusted accordingly would still fall within the mild/moderate risk range for psychological distress.

Our study was exploratory as comparative assessment of similar ethnic groups in Australia and New Zealand has not previously been attempted, however, there are limitations that need to be acknowledged. Firstly, our desire to use pre-translated, culturally validated instruments in Farsi considerably limited the choice of instruments available. None of the specialised refugee instruments was available in Farsi, nor were we able to locate any other commonly used tools in that language when the study was developed in 2006-7. Some authors prohibit independent translation, and as it was beyond the scope of our study to undertake a full translation/validation procedure, a key selection criterion was suitable instrument language availability. However, because these instruments have not commonly been used with refugee groups, comparison data is limited. Although validation should ideally be carried out with each target group, we had to rely on those who translated the instruments and used Farsi-speaking groups as a proxy for our Afghan and Kurdish participants. The chain referral sampling method used also limits generalisability of our results to a wider population, although the personal endorsements characterised by this method helped break down barriers, providing reassurance to potentially suspicious participants. This proved particularly helpful for recruitment of female participants and helped ensure a large enough sample for a valid study.

## Conclusion

Overall, our experience with these three instruments, the Kessler-10 Psychological Distress scale, General Perceived Self Efficacy scale and Personal Well-Being Index suggests they are suitable for use with former refugees from the Middle East and Afghanistan. They were easy to obtain in appropriate languages and scripts, generally presented no significant problems for participant completion, have population datasets available for comparison and showed good reliability when tested with our sample. The majority of Afghan participants completed Farsi language versions with most Kurdish participants preferring English questionnaires to Farsi, and no participant choosing Arabic or Turkish versions. Participants settled 11 years or longer were more likely to complete English versions than those settled ten years or less, so provision of study materials in suitable language translations for participants within this time frame is important.

Despite predictions of an increasing number of refugees in the future, at present there are limited methodological articles available to assist researchers planning studies with ethnic minority groups. Reviews of suitable instruments to allow collection of consistent and comparable data from refugees is needed. As our societies become increasingly multicultural, there is an imperative to ensure research with diverse ethnic groups is robust and conceptually sound, so instrument evaluation, cross-cultural and linguistic preferences, and interpretation of results should all be taken into consideration as part of the research process.

## Competing interests

The authors declare that they have no competing interests.

## Authors' contributions

CS-H conceived the study, participated in its design, co-ordination and data collection, and drafted the manuscript. ST participated in the design of the study and helped draft the manuscript. Both authors read and approved the final manuscript.
